# Optimization of tumor-treating field therapy for triple-negative breast cancer cells in vitro via frequency modulation

**DOI:** 10.1186/s12935-023-02959-x

**Published:** 2023-06-07

**Authors:** Austin R. Smothers, Jason R. Henderson, John J. O’Connell, Jonathan M. Stenbeck, Delphine Dean, Brian W. Booth

**Affiliations:** 1 Center for Innovative Medical Devices and Sensors (REDDI Lab), Clemson University, Clemson, SC USA; 2 Department of Bioengineering, Clemson University, Clemson, SC USA; 3 Quiverent LLC, Greenville, SC USA; 4 Prisma Health Cancer Institute, Prisma Health, Greenville, SC USA; 5 Clemson University School of Health Research, Clemson, SC USA; 6 University of South Carolina School of Medicine-Greenville, Greenville, SC USA

**Keywords:** Breast cancer, Electric field intensity, Frequency modulation, Oscillating electric fields, Radiotherapy, Triple-negative breast cancer (TNBC), Tumor-treating fields (TTFields)

## Abstract

**Purpose:**

Currently, tumor-treating field (TTField) therapy utilizes a single “optimal” frequency of electric fields to achieve maximal cell death in a targeted population of cells. However, because of differences in cell size, shape, and ploidy during mitosis, optimal electric field characteristics for universal maximal cell death may not exist. This study investigated the anti-mitotic effects of modulating electric field frequency as opposed to utilizing uniform electric fields.

**Methods:**

We developed and validated a custom device that delivers a wide variety of electric field and treatment parameters including frequency modulation. We investigated the efficacy of frequency modulating tumor-treating fields on triple-negative breast cancer cells compared to human breast epithelial cells.

**Results:**

We show that frequency-modulated (FM) TTFields are as selective at treating triple-negative breast cancer (TNBC) as uniform TTFields while having a greater efficacy for combating TNBC cell growth. TTField treatment at a mean frequency of 150 kHz with a frequency range of ± 10 kHz induced apoptosis in a greater number of TNBC cells after 24 h as compared to unmodulated treatment which led to further decreased cell viability after 48 h. Furthermore, all TNBC cells died after 72 h of FM treatment while cells that received unmodulated treatment were able to recover to cell number equivalent to the control.

**Conclusion:**

TTFields were highly efficacious against TNBC growth, FM TTFields showed minimal effects on epithelial cells similar to unmodulated treatment.

## Introduction

Triple-negative breast cancer (TNBC) makes up ~ 10–20% of breast cancers in women and is the most aggressive form of breast cancer [1–3]. TNBC is highly resistant to hormone therapy due to its lack of expression of three characteristic receptors: estrogen receptor (ER), progesterone receptor (PR), and human epidermal growth factor receptor 2 (HER2). Due to a lack of effective therapies against TNBC, some groups have investigated low-energy, low-frequency oscillating electric fields to combat disease progression in vitro [4].

Oscillating electric field (OEF) treatment is effective against brain, lung, and ovarian tumor cell populations both in vitro and in vivo [5-9]. When exposed to OEF during mitosis, nuclear components of cells such as microtubules, spindle fibers, and chromosomes act as dipoles and point charges. These components become misaligned during the metaphase-anaphase transition as a result of OEF treatment, causing cellular stress. This cellular stress leads to mitotic exit or early apoptosis, which decreases cellular proliferation [10, 11]. More specifically, TTFields target septin protein complexes involved in microtubule stabilization to mediate mitotic disruption [10].

Tumor-treating fields (TTFields) represent a very narrow range of OEF [6]. Studies of TTFields in varying cancer cell lines have exclusively attempted to determine a discrete frequency at which maximal cell death is achieved for glioblastoma multiforme (GBM), non-small cell lung cancer (NSCLC), ovarian cancer, and pancreatic cancer [6, 7, 12–16]. Human breast carcinoma cells have also been investigated and are most affected by field frequencies of 150 kHz with a field intensity of 1.75–3 V/cm [4, 7]. While results for in vitro research and clinical trials exhibited some moderate success in disease stability and patient survival, patients that participated in clinical trials received treatment for up to 20 h a day every day [17, 18] and received some form of conjunctive chemotherapy or radiotherapy [13–15, 17, 18]. The extended length of treatment required for success may indicate that optimal modes of treatment for TTFields do not currently exist, and the inclusion of conjunctive therapies for disease treatment makes it unclear how much TTFields contributed to disease stability. The lack of a single optimal treatment frequency may be the result of dipole moments of dividing cells changing due to differences in cell size, shape, and ploidy during later stages of mitosis [6]. Thus, a singular treatment frequency for maximal cell death may not exist for an entire heterogeneous cell population. A more dynamic TTField delivery method may be a more effective treatment strategy.

Due to the heterogeneity and distribution of cell size and ploidy during mitosis [19], we anticipate that the range of dipole moments of cancer cells is also heterogeneous during different phases of cell growth. This would be compounded by intra-tumor heterogeneity, especially in breast cancer [20]. Incidentally, while a single frequency may be ideal for the modal average dipole moment in a distribution of cells, we propose modulating the frequency of the carrier wave is a more effective strategy. Additionally, as cells adapt to a continuous delivery of TTFields, the ideal treatment frequency for maximal cell death changes over time [21]. This is likely due to changes in size and ploidy of the adapting cells. Consequently, we posit that cells may be less able to adapt to TTFields delivered over a spectrum of frequencies rather than a single continuous frequency. Our treatment strategy is to tune the average current frequency to the modal dipole moment of the distribution of cells while utilizing modulation to cover a range of frequencies, thus allowing for adjusted treatment for heterogeneous tumor cell populations.

## Methods and materials

### Cell culture parameters

MDA-MB-231 (human TNBC) cells were grown in DMEM plus 10% fetal bovine serum (FBS) and 1% antibiotic-antimycotic (anti-anti) in a CO_2_ incubator (5% CO_2_) at 37 °C. MCF-12 (human breast epithelial) cells were grown in DMEM with MEGM Growth Supplements Singlequots™, plus 10% FBS and 1% anti-anti at the same incubator conditions. 484 mm [2] plastic microscope coverslips were sterilized using 70% ethanol and placed into each well of a 6-well cell culture plate (Corning Inc., Corning, NY) as performed previously [4]. Cells were seeded at ~ 1 × 10^5^ cells per well onto each coverslip, and additional cell culture media was added to achieve a 2 mL total volume solution per well. Cells were given 24–48 h after seeding to adhere before starting treatment.

Co-cultures of MDA-MB-231 and MCF-12 cells were grown in DMEM cell culture media with MEGM Growth Supplements Singlequots™, plus 10% FBS and 1% anti-anti media at the same incubator conditions as previously described. Sterilizing and seeding techniques are also equivalent, however, cells were seeded at a 1:1 ratio with a total of ~ 1 × 10^5^ cells per well.

### Lentiviral transfection and cell staining

Lentiviral transfection was performed as previously reported (Park et al.) [22]. MCF-12 cells were seeded into 96-well plates and grown under normal culture conditions (5% CO_2_ at 37 °C) until 100% confluent. Cell culture media was removed and replaced with antibiotic-free media and cultures continued to grow overnight. Cells were washed with PBS, and 30 µL of antibiotic-free media plus 20 µL of Cignal Lenti Reporter (Qiagen, Hilden, Germany) were added to each well. MCF-12 cells were transfected with green fluorescent protein (GFP) particles. Cells were then incubated for 24 h under normal culture conditions. Media was removed from each well and 100 µL of fresh media containing 500 ng/mL of puromycin (Life Technologies Corporation, Grand Island, NY) was added for selection of transfected cells. Puromycin-media was replaced every 3 days for 12 days, after which it was replaced with growth media described above.

Membrane staining for MDA-MB-231 and MCF-12 cells were performed using BioTracker NIR570 Cytoplasmic Membrane Dye and BioTracker 655 Cytoplasmic Membrane Dye (Sigma-Aldrich, St. Louis, MO), respectively. For adherent cells, staining media was prepared at 1 µM concentration and 500 µL of staining media were added to wells containing 1 × 10^5^ cells. Cells were incubated for 20 min at 37 °C and then washed three times for 5 min each with regular cell culture media while incubating at normal culture conditions. For suspended cells, cells were suspended at 1 × 10^6^ cells/mL in 10 mL of 5 µM cell labeling solution. Cells were incubated for 20 min at 37 °C and then centrifuged at 1000 rpm for 5 min. Cells were then re-suspended in cell culture media and added to cell culture wells at 1 × 10^5^ cells per well.

DAPI (Invitrogen, Waltham, MA) staining was also performed for MDA-MB-231 and MCF-12 cells. 2 mL of deionized (DI) water was used to create a 14.3 mM DAPI stock solution. 2.1 µL of the stock solution were added to 100 µL of PBS to create a 300 µM dilution, which was used to create a 300 nM DAPI stain solution. Adherent cells were washed 3 times in PBS, and 500 µL of 300 nM DAPI solution were added to the adherent cells. Cells were incubated for 5 min at 37 °C, protected from light. After the stain solution was removed, the cells were washed 3 more times in PBS and then imaged.

Apoptosis quantification was performed as recommended for the ORFLO Moxi GO II by ORFLO [23]. Cells were isolated in suspension and then centrifuged at 1000 rpm for 5 min. Cells were then re-suspended at 1 × 10^6^ cells/mL in Annexin V binding buffer. 100 µL aliquots of 1 × 10^5^ cells were created. 2 µL of FITC—Annexin V conjugate were added, and the cells were gently vortexed and then incubated for 15 min at room temperature, protected from light. 300 µL of Annexin V binding buffer and 2 µL of 1 mg/mL Propidium Iodide (PI) were added, and then each tube was incubated for 5 min at room temperature. Finally, 8 µL of ORFLO Flow Reagent were added to each tube, and flow cytometry was performed to detect apoptosis.

### Delivery of frequency-modulated TTFields in vitro

A function generator (Tektronix AFG1062, Tektronix, Inc., Beaverton, OR) is connected to a 4-channel relay module to create the desired waveforms and magnitudes of modulation to be delivered to cells within a 6-well cell culture plate as performed elsewhere [4]. An Arduino control board (Arduino Uno Rev3 SMD, Arduino, New York, NY) is also connected to the 4-channel relay module to control multiple simultaneously alternating waveforms, their commutation times between parallel plate pairs, and the time delay between commutations. Finally, the relay module is connected to paired stainless steel electrodes fitted into a 6-well cell culture plate lid via laser-etched slots. Furthermore, the electrodes are held in place by 3D-printed high-temperature resin channels (Form 2 3D Drucker, Formlabs, Somerville, MA). Waveforms were monitored using a Tektronix TBS 2000 Series Digital Oscilloscope (Tektronix, Inc., Beaverton, OR). Sterilization and cleaning of machinery and other equipment is detailed in previous experiments [4].

Using this setup, we have conducted frequency-modulated (FM) TTField treatment on TNBC cells and breast epithelial cells to compare treatment effects on differing cell lines. Cells were subjected to 24, 48, and 72 h of uninterrupted TTField treatment. Flow cytometry data and microscope images at 24-, 48-, and 72-hour time points were compared against unmodulated (UM) TTField treatment, a negative control group that received no treatment, and a positive control group that received 400 µM Cisplatin treatment. Fields were delivered at 1.5 V/cm electric field intensity with a mean carrier frequency of 150 kHz and frequency modulation was utilized at 10 kHz deviation over a modulating period of 2 min. Further details on TTField device parameters can be found with previous experiments [4].

### Chemotherapy delivery

400 µM Cisplatin was used as a positive control for cell death and induced apoptosis. A stock solution of 10 mM Cisplatin (Sigma-Aldrich, St. Louis, MO) was created using 140 mM NaCl-PBS solution and 100 mg of Cisplatin powder. 800 µM dilutions were created in cell culture media, and 1 mL of Cisplatin-media solution was added to cell culture well already containing adherent cells and 1 mL of fresh cell culture media to cut the concentration in half, resulting in 400 µM Cisplatin solutions.

### Microscopy and flow cytometry

Brightfield microscope images were taken using a Zeiss® Axiovert 40 CFL inverted fluorescence phase contrast microscope and Axiovision Rel. 4.8 imaging software (Carl Zeiss Industrial Metrology, Maple Grove, MN). An ORFLO® Moxi Go II flow cytometer (ORFLO Technologies, Ketchum, ID) with single-channel cassettes was used to perform cell size and viability counts. When taking viable cell counts using flow cytometry, data for each cell line were gated based on the measured mean diameter of the cell ± three standard deviations of the mean based on a sample size of *n* = 10 from experimentation during device validation in previous experiments [4]. Average diameter for MDA-MB-231 cells were further validated by comparison to previous studies [24], but reliable previous data for MCF-12 cells could not be found. Finally, all cell counts for all time points were normalized based on an exact initial seeding of 1 × 10^5^ cells per well for ease of data interpretation and comparison.

### Scratch test

MDA-MB-231 and MCF-12 cells were seeded at 100,000 cells per well and allowed to grow to confluence (~ 72 h after initial seeding). A 200 µL micropipette tip was used to create a “wound” within each culture (*n* = 3), and the initial diameter of the wound was measured and recorded in three different locations using EVOS FL Auto PearlScope64 imaging software (Life Technologies, Carlsbad, CA), and then averaged. TTField treatment was given to the cells for 24 h, and the diameter of the wound was measured, recorded, and averaged once more. Data is presented as percent change in diameter.

### Data interpretation and statistical analysis

For all quantitative data, a standard one-way ANOVA and unpaired *t*-test were performed to determine statistical significance. For determining statistical significance for apoptotic cell percentages, samples were compared to positive control. For determining statistical significance for percent viable cells to control, samples were compared to the negative control.

## Results

### Treatment efficacy after 24 h by modulating TTField frequency

**Fig. 1:**
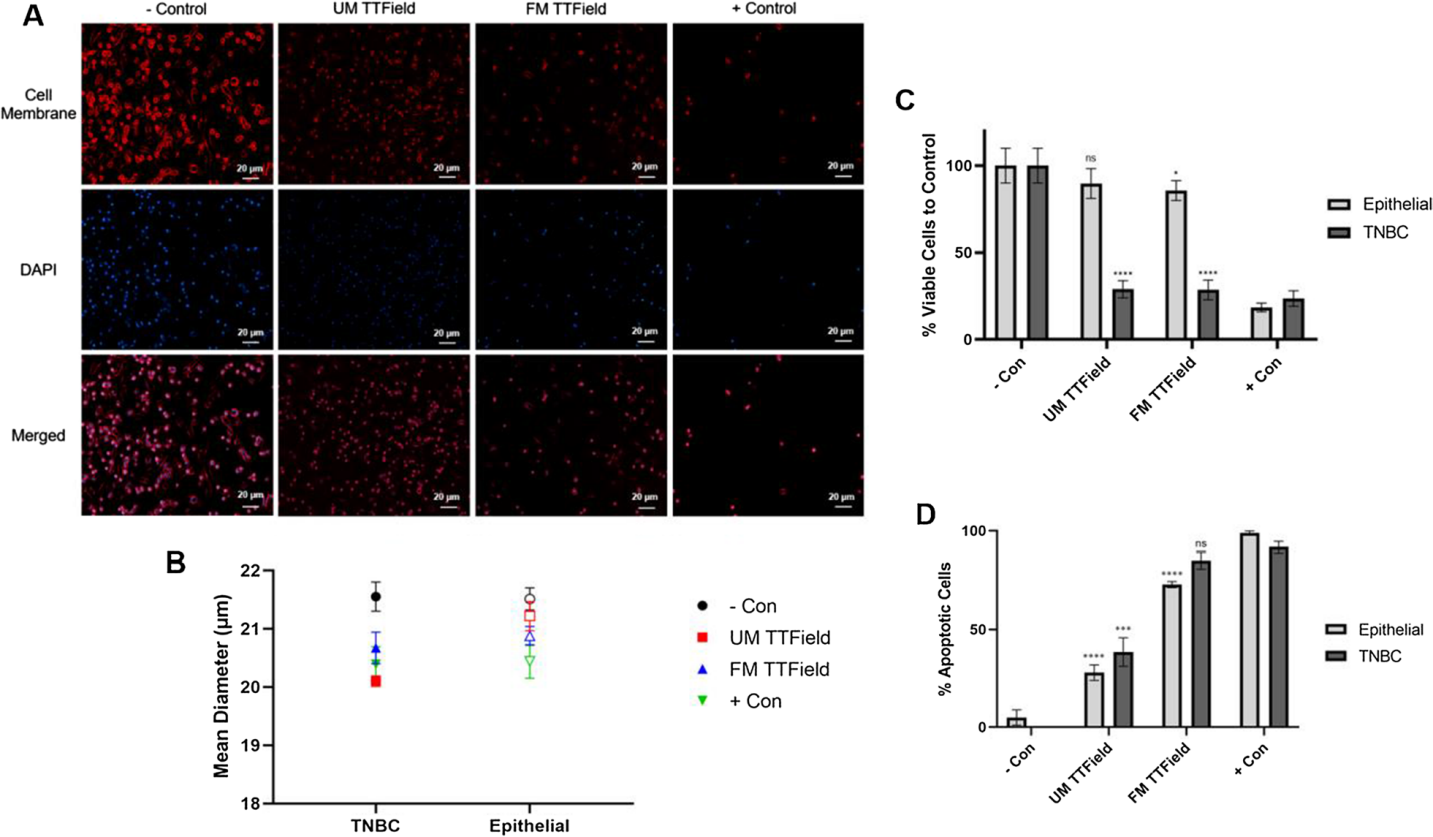
Treatment of TNBC and epithelial cells via unmodulated (UM) and frequency-modulated (FM) TTFields after 24 h. **A** TNBC cells were stained with 655 Cytoplasmic Membrane Dye and DAPI. After 24 h, UM and FM TTFields show significant damaging effects. **B** TNBC cells also showed consistent decrease in average diameter after 24 h of UM and FM TTField treatment while epithelial cells did not experience as significant of changes. **C** Percent viable cells and **D** percent apoptotic cells for TNBC and epithelial cells were recorded after 24 h. UM and FM TTFields show significantly decreased numbers of viable cells and FM TTFields show increased rates of apoptosis within TNBC cells. (*P* < 0.05 = *, *P* < 0.01 = **, *P* < 0.001 = ***, *P* < 0.0001 = ****) (Scale bars = 20 μm)

Total cell counts (*n* = 5) and apoptotic cell counts (*n* = 3) for both TNBC (MDA-MB-231) and breast epithelial (MCF-12) cells that received no treatment (negative control), UM TTField treatment, FM TTField treatment, and 400 µM Cisplatin (positive control) were recorded after 24 h (Fig. 1). When subjected to either UM or FM TTFields, TNBC cell populations decreased to ~ 29% of negative control (*P* < 0.0001) (Fig. 1C). Conversely, breast epithelial cells only decreased to 89.76% of the negative control when subjected to UM treatment (*P* = 0.1010) and 85.70% of the negative control when subjected to FM treatment (*P* = 0.0171).

FM TTFields showed greater efficacy than UM TTFields for inducing apoptosis after 24 h of uninterrupted treatment: 38.47% of TNBC cells showed signs of apoptosis under UM TTFields (*P* = 0.0003) while 85.12% of TNBC cells showed signs of apoptosis under FM TTFields (*P* = 0.0906) as compared to the positive control which induced apoptosis in 91.76% of cells (Fig. 1D). Furthermore, following 24 h of TTField treatment, TNBC cell diameter was 1.45 μm (UM, *P* < 0.0001) and 0.88 μm (FM, *P* = 0.0007) smaller than negative control, whereas epithelial cell diameters were 0.29 μm greater (UM, *P* = 0.0728) than negative control and 0.34 μm (FM, *P* = 0.0336) smaller than negative control (Fig. 1B). The significant decrease in cell diameter in TNBC cells could be an indication of early apoptosis.

### Treatment efficacy after 48 and 72 h by modulating TTField frequency

**Fig. 2:**
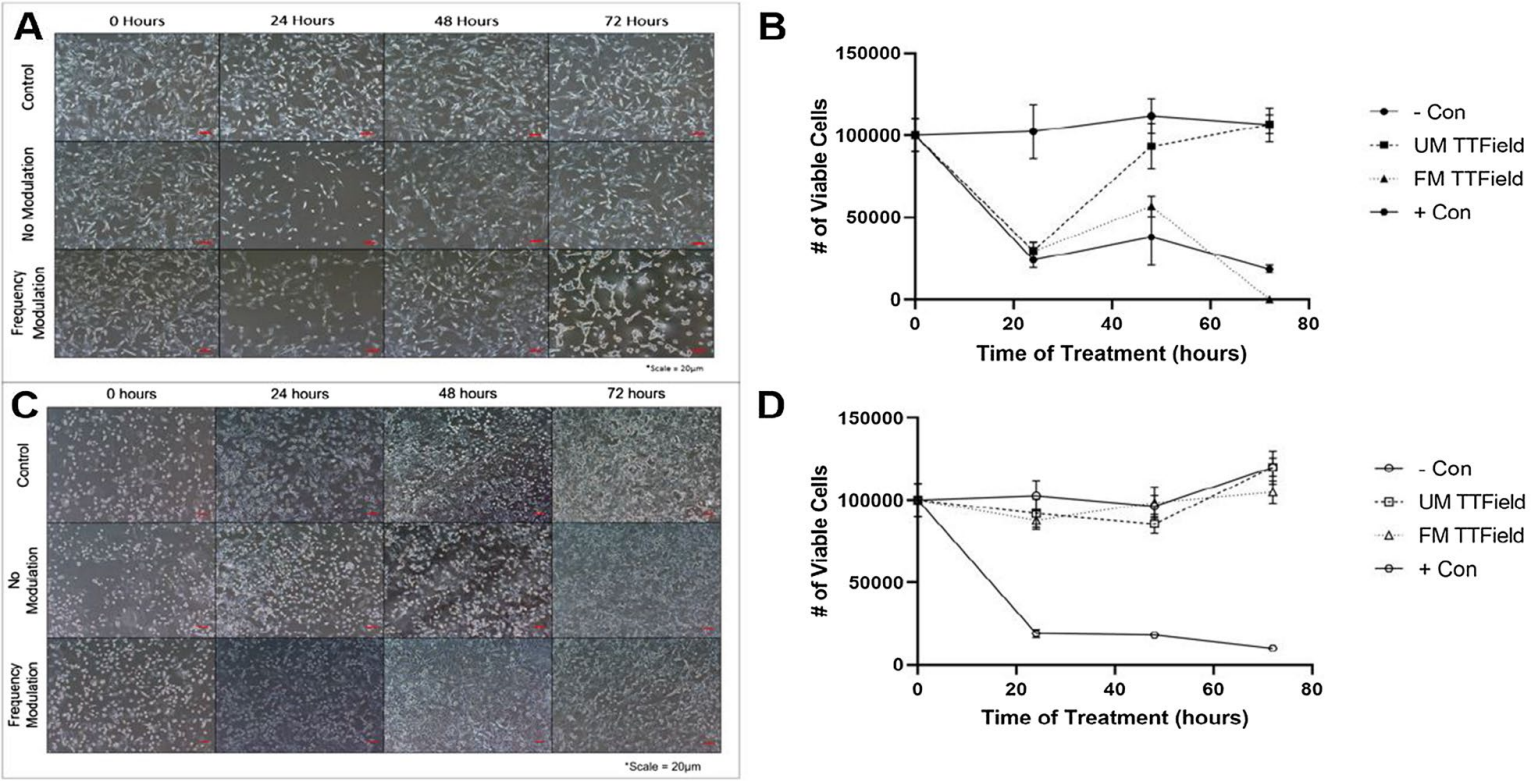
Treatment of TNBC and epithelial cells with UM and FM TTFields over 72 h. **A** Images of TNBC cells show qualitative evidence of decrease in cell numbers after 24 h, recuperation through 48 h, and explicit signs of blebbing and compromised cell membranes through 72 h as a result of FM TTFields [4]. **B** Quantitative viable cell counts show that UM TTFields were ineffective at sustaining cell death in TNBC cells through 72 h, but FM TTFields were extremely effective at eliminating TNBC cells. **C** Epithelial cells showed no signs of morphology changes through 72 h as a result of any TTField treatment. **D** Both UM and FM TTField treatment had no significant effect on epithelial cell growth as compared to negative control at 24, 48, or 72 h. (Scale = 20 μm)

After 48 h of UM treatment, TNBC cells began acclimating to treatment, recovering to ~ 74% of control cell count (*P* = 0.0015) (Fig. 2A, B) while breast epithelial cells showed some signs of cell death from treatment, being ~ 89% of negative control cell count (*P* = 0.0252) (Fig. 2C, D). After 72 h of UM treatment, TNBC and breast epithelial cells showed no signs of influence by the treatment, having congruent cell counts (*P* > 0.9999) to negative control for both cell lines (Fig. 2).

FM TTField treatment for TNBC after 48 h prevented cells from recovering as quickly as they did while receiving UM treatment; cell counts were only ~ 51% (*P* < 0.0001) of negative control as compared to the previously mentioned 74% (Fig. 2A, B). Conversely, breast epithelial cells that received 48 h of FM treatment had cell counts of ~ 102% of negative control (*P* = 0.6790) (Fig. 2C, D). Finally, after 72 h of FM treatment, TNBC cells showed major signs of blebbing and compromised cell membranes. When cells were trypsinized for flow cytometry, no cells could be observed qualitatively (microscope imaging) or quantitatively (flow cytometry), thus making cell counts zero (*P* < 0.0001) (Fig. 2A, B). Epithelial cells did not show any signs of damage after 72 h of treatment but did have only ~ 88% of cells compared to negative control (*P* = 0.0050) (Fig. 2C, D).

### FM TTField efficacy against co-cultured TNBC and epithelial cells

**Fig. 3:**
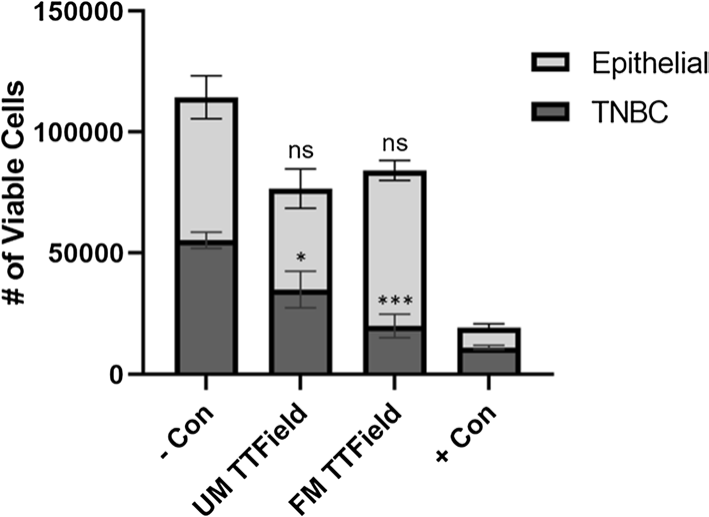
Treatment of co-cultured TNBC and epithelial cells with UM and FM TTFields after 24 h. UM and FM TTFields have similar effect trends on individually cultured and co-cultured TNBC and epithelial cells. However, co-cultured cells experienced less cell death than individually cultured cells, especially from UM TTFields. (*P* < 0.05 = *, *P* < 0.01 = **, *P* < 0.001 = ***, *P* < 0.0001 = ****)

 Co-cultures of TNBC and breast epithelial cells were treated with either UM or FM TTFields as a more accurate representation of clinical treatment (*n* = 3). After 24 h of treatment using either UM or FM TTFields, mono-cultured and co-cultured cells displayed a very similar trend in treatment response for both cell lines (Fig. 3). However, co-cultured TNBC and epithelial cells did not seem to be as affected by UM TTFields (TNBC, *P* = 0.0138; epithelial, *P* = 0.0655) or FM TTFields (TNBC, *P* = 0.0005; epithelial, *P* = 0.4295) as compared to mono-cultured cells (Fig. 1).

**Fig. 4:**
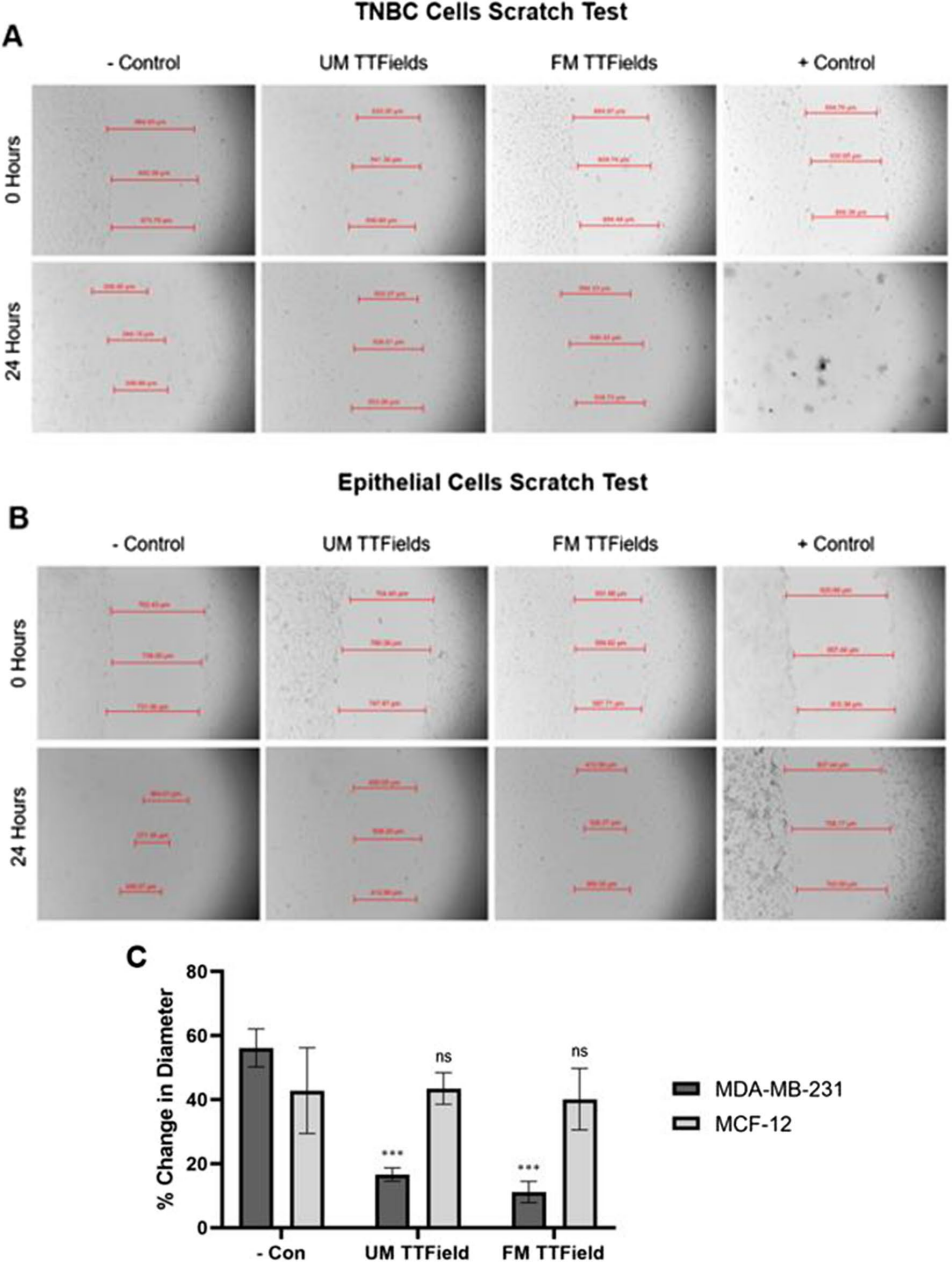
A scratch test for TNBC and epithelial cells after 24 h of UM and FM TTFields treatment. UM and FM TTFields had a similar effect in preventing TNBC cell migration within the wound. This is also true for epithelial cells, although FM TTFields were less consistent in doing so. (*P* < 0.05 = *, *P* < 0.01 = **, *P* < 0.001 = ***, *P* < 0.0001 = ****)

### Scratch test

A scratch test was also performed for both TNBC and breast epithelial cells (*n* = 3). After 24 h, TNBC cells negative control wound diameter decreased by approximately 56%, UM TTField-treated wound diameter decreased by 17% (*P* = 0.0004), and FM TTField-treated wound diameter decreased by only 11% (*P* = 0.0003) (Fig. 4). Epithelial cells were able to close the gap much more adequately after both UM and FM treatment, having a wound diameter decrease of 43% (*P* = 0.9373) and 40% (*P* = 0.7952), respectively. This is compared to the negative control which also had a wound diameter decrease of 43%.

## Discussion

Our treatment process via delivery of FM TTFields at an average frequency of 150 kHz modulated over a range of ± 10 kHz shows increased efficacy against TNBC cell growth compared to UM TTFields while maintaining a high level of selectivity. This is supported by the fact that, after 48 h of treatment, TNBC cells did not acclimate to FM TTFields as well as they did for UM TTFields. In fact, after 72 h of FM TTField treatment, there were no observable viable TNBC cells (Fig. 2). We believe that the increased efficacy of FM TTFields is largely due to being able to cover a spectrum of frequencies to address heterogeneous tumor populations while also preventing cells to become desensitized to the treatment as it is always changing.

Despite the increased efficacy of FM TTFields against in vitro TNBC growth, treatment can still be improved. For example, it is unclear what range of frequencies should be used for maximum efficacy. We chose 10 kHz as it does not significantly deviate from the “optimal” frequency for maximal TNBC cell death (150 kHz), but the deviation also does not seem to encompass the treatment frequency that most affects breast epithelial cells. Furthermore, we believe that minimizing the deviation will allow for more treatment time to occupy the targeted cell line’s optimal treatment frequency for maximal cell death.

Our investigation also has some limitations. First, only one TNBC cell line was used. In previous experimentation, MDA-MB-231 and HCC38 cells showed differing treatment responses based on electric field intensity [4]. Also, using non-culture treated microscope coverslips may have affected cell adherence and growth, however we did not observe this issue. FM TTField treatment could also produce differing results between cell lines. Furthermore, treatment for three-dimensional cultures could provide a better representation for in vivo treatment than two-dimensional mono- or co-cultures. This would also allow for investigation into metastasis via migration assays. Finally, our setup is limited to treating only two 6-well cell culture plates at one time, limiting treatment throughput for biological and technical replicates.

Amplitude modulation (AM) may also be a viable treatment delivery method to address tumor heterogeneity. We performed experimentation on TNBC cells using AM TTFields and had some initial success but were unable to produce repeatable results (data not shown). However, this could be due to an incompatible device setup, as our machine was unable to support the input voltage necessary for delivering TTFields at greater amplitudes. Also, based on previous experimentation [4, 7], field amplitudes should remain within 1.5–3.0 V/cm, as this showed the greatest treatment selectivity for TNBC cells without destroying epithelial cells.

There are many additional TTField parameters that should be investigated. We believe the most promising factor with this method of treatment is modulation period; it is unclear if modulating frequency or amplitude over a span of minutes or hours or days is the most efficacious. Presumably, modulating over a shorter period will minimize the cells’ ability to become desensitized to treatment.

## Data Availability

All data generated or analyzed during this study are included in this published article.
